# Supplementary material for: The causal relationship between the human gut microbiota and pyogenic arthritis: a Mendelian randomization study
Source: Front Cell Infect Microbiol. 2024 Nov 26;14:1452480. doi: 10.3389/fcimb.2024.1452480 (PMC11629706; doi:10.3389/fcimb.2024.1452480)
Supplement: Supplementary file 1 [file DataSheet1.zip › mendelian test/ebi-a-GCST90027606.csv_leave_one_out.pdf]

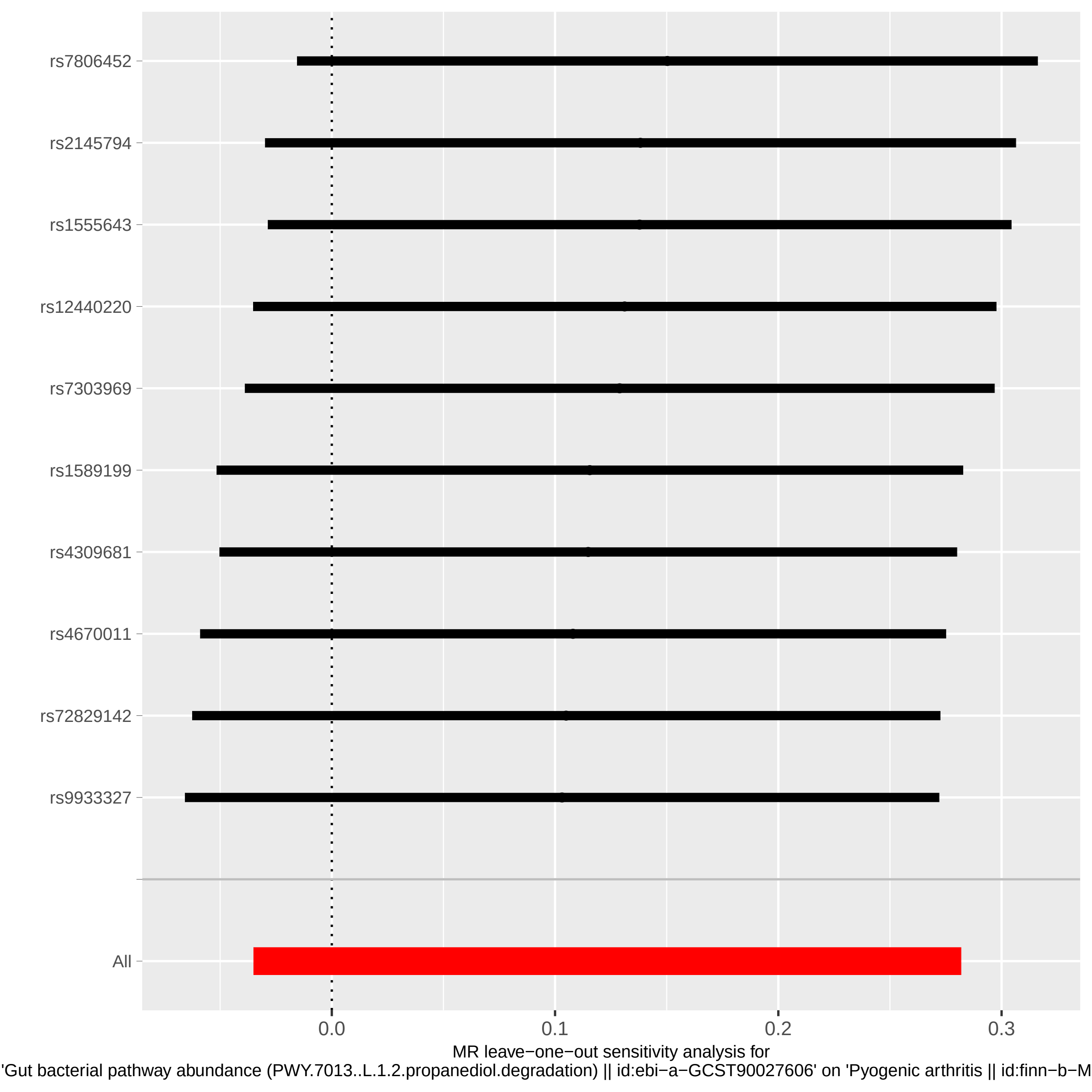

rs7806452
rs2145794
rs1555643
rs12440220
rs7303969
rs1589199
rs4309681
rs4670011
rs72829142
rs9933327
All
0.0
0.1
0.2
0.3
MR leave−one−out sensitivity analysis for
'Gut bacterial pathway abundance (PWY.7013..L.1.2.propanediol.degradation) || id:ebi−a−GCST90027606' on 'Pyogenic arthritis || id:finn−b−M
